# Detecting *TP53* mutations in diagnostic and archival liquid-based Pap samples from ovarian cancer patients using an ultra-sensitive ddPCR method

**DOI:** 10.1038/s41598-019-51697-6

**Published:** 2019-10-29

**Authors:** Nicolai Skovbjerg Arildsen, Laura Martin de la Fuente, Anna Måsbäck, Susanne Malander, Ola Forslund, Päivi Kannisto, Ingrid Hedenfalk

**Affiliations:** 1 Division of Oncology and Pathology, Department of Clinical Sciences, Skåne University Hospital, Lund University, Lund, Sweden; 2 Department of Clinical Pathology, Division of Laboratory Medicine, Skåne University Hospital, Lund, Sweden; 3 Department of Medical Microbiology, Laboratory Medicine Region Skåne, Lund University, Lund, Sweden; 4 Department of Obstetrics and Gynaecology Lund, Skåne University Hospital, Lund University, Lund, Sweden

**Keywords:** PCR-based techniques, Ovarian cancer, Cancer prevention, Ovarian cancer

## Abstract

High-grade serous ovarian cancer (HGSOC) is the most common subtype of epithelial ovarian cancer and early detection is challenging. *TP53* mutations are a hallmark of HGSOC and detection of these mutations in liquid-based Pap samples could provide a method for early diagnosis. Here we evaluate the use of IBSAFE, an ultra-sensitive droplet digital PCR (ddPCR) method, for detecting *TP53* mutations in liquid-based Pap samples collected from fifteen women at the time of diagnosis (diagnostic samples) and/or up to seven years prior to diagnosis (archival samples). We analysed tumours for somatic *TP53* mutations with next generation sequencing and were able to detect the corresponding mutations in diagnostic samples from six of eight women, while one patient harboured a germline mutation. We further detected a mutation in an archival sample obtained 20 months prior to the ovarian cancer diagnosis. The custom designed IBSAFE assays detected minor allele frequencies (MAFs) with very high assay sensitivity (MAF = 0.0068%) and were successful despite low DNA abundance (0.17–206.14 ng, median: 17.27 ng). These results provide support for further evaluation of archival liquid-based Pap samples for diagnostic purposes and demonstrate that ultra-sensitive ddPCR should be evaluated for ovarian cancer screening in high-risk groups or in the recurrent setting.

## Introduction

While the incidence and mortality of cervical cancer have decreased radically since the introduction of the Papanicolaou test (Pap test)^1–3^, overall survival from ovarian cancer has not changed substantially over the past 50 years^4^. High-grade serous ovarian cancer (HGSOC) confers an overall 5-year survival rate around 45%, but outcomes vary greatly between disease stages, with 5-year survival rates above 70% in stage I and II disease. However, symptoms of HGSOC often present in late stages (III and IV) of the disease, resulting in 5-year survival rates between 26–42%^5^. None of the approaches aimed at early detection, including serum CA-125 and trans-vaginal ultrasound have been successfully applied in a screening setting due to limited specificity and sensitivity^6–8^.

HGSOC is believed to arise in the fallopian tube epithelium^9^, and mutations in the tumour suppressor gene *TP53* are believed to be a very early event in the carcinogenesis of HGSOC^10^. A recent study by Labidi-Galy *et al*. (2017) showed shared *TP53* mutations in patient-matched pre-cancerous and cancerous lesions (including so-called p53 signatures, Serous Tubal Intraepithelial Carcinoma (STIC) lesions and invasive carcinomas) from nine patients with HGSOC, providing support for the possibility of discovering tumour-driving mutations in early stages of the disease^11^. Apart from frequent mutations in *TP53*, these cancers are characterised by few recurrent mutations and instead harbour wide-spread chromosomal instability^12^. Recent studies have investigated chromosomal instability and levels of somatic copy number alterations as a prognostic tool and therapeutic target in HGSOC^13,14^.

A promising development occurred in 2013, when Kinde *et al*. showed that somatic mutations in DNA shed from endometrial and ovarian cancers could be detected in standard liquid-based Pap test specimens by massively parallel sequencing^15^. While highly sensitive for endometrial cancer, the method was not able to detect more than 41% of ovarian cancers using a panel of 12 genes commonly mutated in these tumours. Subsequent studies from this research group in collaboration with others have attempted to increase the sensitivity for detection of ovarian cancer by introducing new procedures including lavage of the uterine cavity^16^, by combining Pap test and plasma sampling and by complementing the mutation assay with an assay for aneuploidy^17^. However, clinical sensitivity remains a challenge with this approach, and will require extensive modelling before application in clinical diagnostics^18–20^. Moreover, these approaches have only been applied in symptomatic patients, at the time of diagnosis, and have so far not been evaluated in pre-symptomatic women prior to the time of diagnosis.

Droplet Digital PCR (ddPCR) provides an alternative to sequencing-based methods, with the advantages of increased sensitivity, rapid turnover time and ease of use^21^. Analysis of circulating tumour DNA using ddPCR has shown great potential for prognostication and monitoring of treatment response in several tumour types including gynaecological cancers^22–24^.

In this study, we analysed liquid-based archival Pap samples (archival samples) from fifteen women collected approximately two to seven years before they were diagnosed with HGSOC, and from nine of these women also liquid-based diagnostic Pap samples (diagnostic samples) collected at the time of the HGSOC diagnosis. Mutations in *TP53* were identified by next generation sequencing (NGS) of tumour tissue obtained at surgery. We used the ultra-sensitive ddPCR IBSAFE technology for mutation detection in Pap samples and used a commercially available approach from Bio-Rad as a control where applicable. The analysis of liquid Pap samples from pre-symptomatic women subsequently diagnosed with HGSOC has, to our knowledge, not been previously reported.

## Results

### Patient cohort

**Figure 1:**
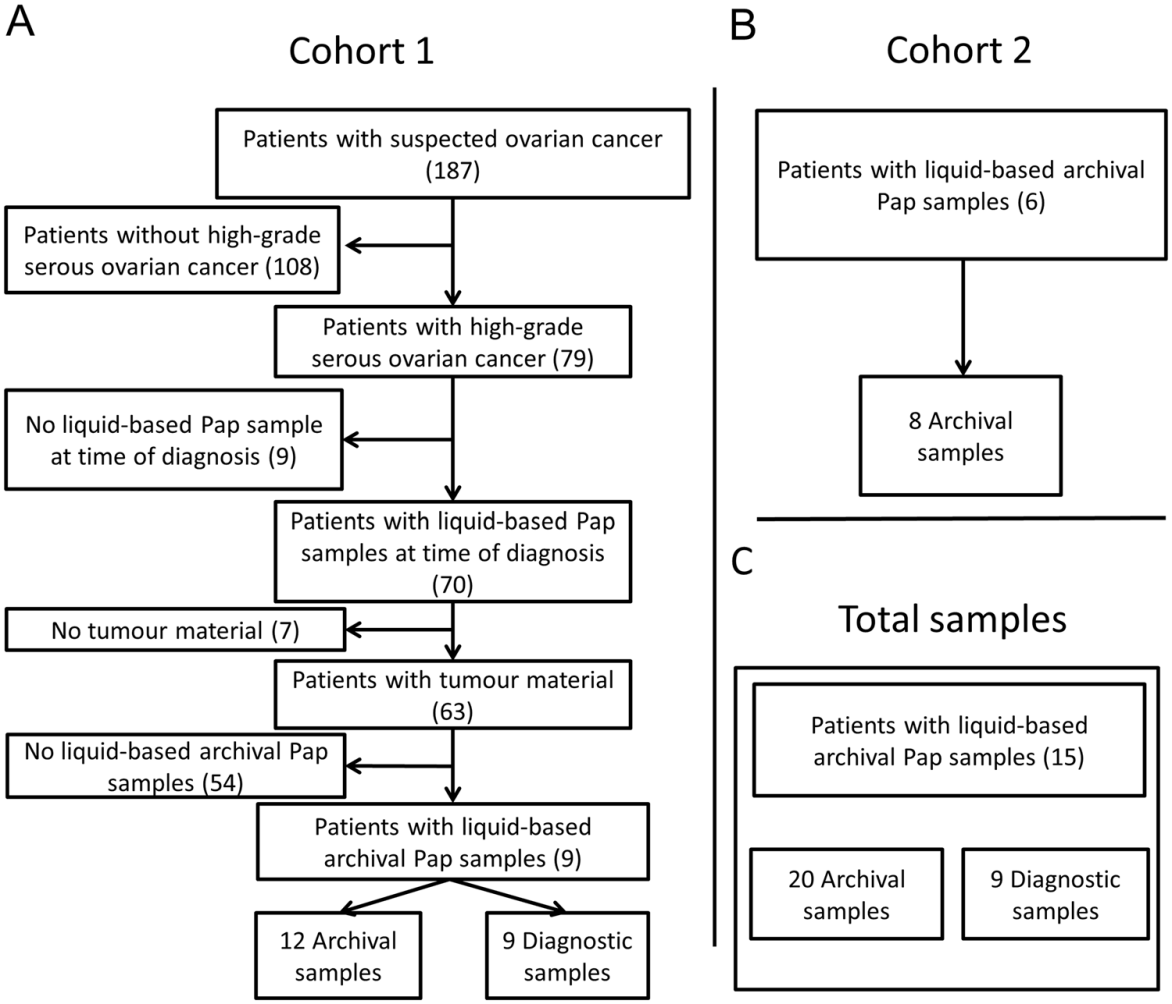
Flowchart of patients included in the study for cohort 1 (**A**), cohort 2 (**B**), and the total number of samples included in this study (**C**).

A total of 20 archival samples were obtained from 15 patients from cohorts 1 and 2 (Fig. 1A–C). Fresh frozen tumour tissue was available from 11 patients, while FFPE tumour tissue was available from four patients. Corresponding blood was available from 14 patients, while nine patients from cohort 1 had diagnostic samples. Patient characteristics are provided in Table 1. The time from the collection of archival samples to the time of HGSOC diagnosis ranged from 20 to 95 months; two patients had more than one archival sample and the remaining patients had a single archival sample. DNA concentrations in diagnostic and archival samples varied between 5.2–55.2 ng/µl (median 16.3 ng/µl) and 0 (too low)-19.4 ng/µl (median 2.755 ng/µl) respectively but were not linked to disease stage. DNA concentrations in archival samples were lower than in diagnostic samples (P = 0.02).

**Table 1:** Patient characteristics.

Patient		Stage*	Tumour DNA concentration (ng/µl)	Diagnostic sample DNA concentration (ng/µl)	Number of archival samples	Time between archival and diagnostic sample (months)	Archival sample DNA concentration (ng/µl)
1		IIIC	113.4^†^	33.5	1	51	Too low
			164.8^‡^				
2	A	IVB	547.8	10.7	4	88	7.6
	B					77	Too low
	C					66	1.16
	D					53	1.21
3		IVB	366.8	44.4	1	95	9.31
4		IVA	223.6	5.2	1	90	19.4
5		IIA	166.7	26.4	1	81	1.01
6		IIA	326.1	16.3	1	82	0.2
7		IVB	279.6	5.74	1	64	0.72
8		IIB	251.5	55.2	1	75	7.9
9		IIIA	370.7	14.3	1	86	0.18
10		IIIC	288.0	NA	1	50	2.96
11		IVB	253.3	NA	1	51	0.45
12		IIB	48.3**	NA	1	35	3.37
13	A	IIIB	37.4**	NA	3	46	2.55
	B			NA		32	1.12
	C			NA		20	3.74
14		IIIC	25.8**	NA	1	38	15.5
15		IIIC	62.2**	NA	1	34	12.2

*All tumours were staged according to the International Federation of Gynaecology and Obstetrics criteria^33^. Too low: DNA concentration below the detection threshold of the QUBIT HS assay. ^†^Left ovary. ^‡^Right ovary. NA: Not available. **DNA from FFPE samples.

### Tumour sequencing and analysis

Paired tumour and blood samples were analysed for *TP53* mutations using the INVIEW Oncopanel All-in-one from GATC (Supplementary Table S1). One patient lacked a corresponding blood sample; the tumour sequence was therefore analysed using a normal control constructed from five patients (patients 2, 3, 4, 6 and 9) within cohort 1 and two patients from cohort 2 (patients 11 and 14). At least one mutation was identified for each patient using GEMINI and a hard filter minor allele frequency (MAF) cut-off of 5%. Ten missense mutations, two nonsense mutations, and three frameshift deletions were identified. Mutations were dispersed across the *TP53* gene, with no overlap between patients. Patient 4 displayed two mutations in positions adjacent to each other, which was handled as a single mutation in the downstream analysis (Table 2). All mutations but one were previously recorded in COSMIC (Catalogue Of Somatic Mutations In Cancer); however five of the patients scored neutral or NA in the Functional Analysis through Hidden Markov Models (FATHMM)^25^ scoring by GEMINI (GEnome MINIng). MAFs in the tumours ranged between 8–85% with a median of 64% (Table 3).

**Table 2:** Mutation characteristics.

Patient	Protein Change	Mutation Type	CHR	Start Position	End Position	Reference Allele	Variant Allele	COSMIC ID	FATHMM
1	TP53 - V10I	Missense Mutation	17	7579885	7579885	C	T	COSM45361	Neutral
2	TP53 - Q136E	Missense Mutation	17	7578524	7578524	G	C	COSM43767	Pathogenic
3	TP53 - Y163C	Missense Mutation	17	7578442	7578442	T	C	COSM10808	Pathogenic
4	TP53 - P151S	Missense Mutation	17	7578479	7578479	G	A	COSM10905	Pathogenic
4	TP53 - T150T	Synonymous Mutation	17	7578480	7578480	T	A	NA	NA
5	TP53 - C242F	Missense Mutation	17	7577556	7577556	C	A	COSM10810	Pathogenic
6	TP53 - R333fs	Frame Shift Deletion	17	7574030	7574030	G	-	COSM69084	NA
7	TP53 - P273L	Missense Mutation	17	7577120	7577120	C	A	COSM10779	Pathogenic
8	TP53 - C242*	Nonsense Mutation	17	7577555	7577555	G	T	COSM44378	Pathogenic
9	TP53 - E294*	Nonsense Mutation	17	7577514	7577515	C	A	COSM10856	NA
10	TP53 - Q52fs	Frame Shift Deletion	17	7579532	7579533	TG	-	NA	
11	TP53 - C176Y	Missense Mutation	17	7578403	7578403	C	T	COSM10687	Pathogenic
12	TP53 - R213fs	Frame Shift Deletion	17	7578213	7578213	A	-	COSM5016718	NA
13	TP53 - R175H	Missense Mutation	17	7578406	7578406	C	T	COSM10648	Pathogenic
14	TP53 - G244V	Missense Mutation	17	7577550	7577550	C	A	COSM43652	Pathogenic
15	TP53 - G245V	Missense Mutation	17	7577547	7577547	C	A	COSM11196	Pathogenic

FATHMM: Functional Analysis through Hidden Markov Models^25^.

**Table 3:** Minor allele frequencies of droplet digital PCR and NGS results.

Patient		Tumour			Diagnostic samples			Archival samples		
		NGS MAF	IBSAFE MAF	Bio-Rad MAF	IBSAFE MAF	Bio-Rad MAF	IBSAFE DNA analysed (ng)	IBSAFE MAF	IBSAFE DNA analysed (ng)	IBSAFE estimated concentration (ng/µl)
1		0.51	0.55	NA	0.567607	NA	126	0.459440	0.28	0.024
2	A	0.77	0.78	NA	0.005875	NA	146	0.000000	52.34	4.36
	B							0.000000	0.17	0.014
	C							0.000000	12.76	1.06
	D							0.000000	16.43	1.37
3		0.85	0.86	0.86	0.000068	ND	155	0.000000	78.25	6.52
4		0.08	0.08	NA	0.005272	NA	131	0.000000	139.42	11.60
5		0.7	0.75	NA	0.000782	NA	64.6	0.000000	12.80	1.07
6		0.45	0.54	NA	0	NA	118	0.000000	1.85	0.15
7		0.47	0.56	NA	0	NA	117	0.000000	7.71	0.64
8		0.5	0.55	0.54	0.000226	0.000089	124	0.000000	55.65	4.64
9		0.71	0.69	0.70	0.078814	0.017870	103	0.000000	2.17	0.18
10		0.76	0.84	NA	NA	NA	NA	0.000000	26.42	2.86
11		0.72	0.69	NA	NA	NA	NA	0.000000	4.33	0.47
12		0.68	0.59	NA	NA	NA	NA	0.000000	42.73	4.62
13	A	0.63	0.74	NA	NA	NA	NA	0.000000	18.11	1.96
	B			NA				0.000000	14.15	0.94
	C			NA				0.00042	31.75	3.43
14		0.23	0.16	NA	NA	NA	NA	0.000000	206.14	22.30
15		0.64	0.63	NA	NA	NA	NA	0.000000	70.14	7.58

NGS: next-generation sequencing. NA: Not available. ND: Not detectable. MAF: Minor allele frequency.

### Mutation screening in diagnostic and archival samples using IBSAFE and Bio-Rad ddPCR

**Figure 2:**
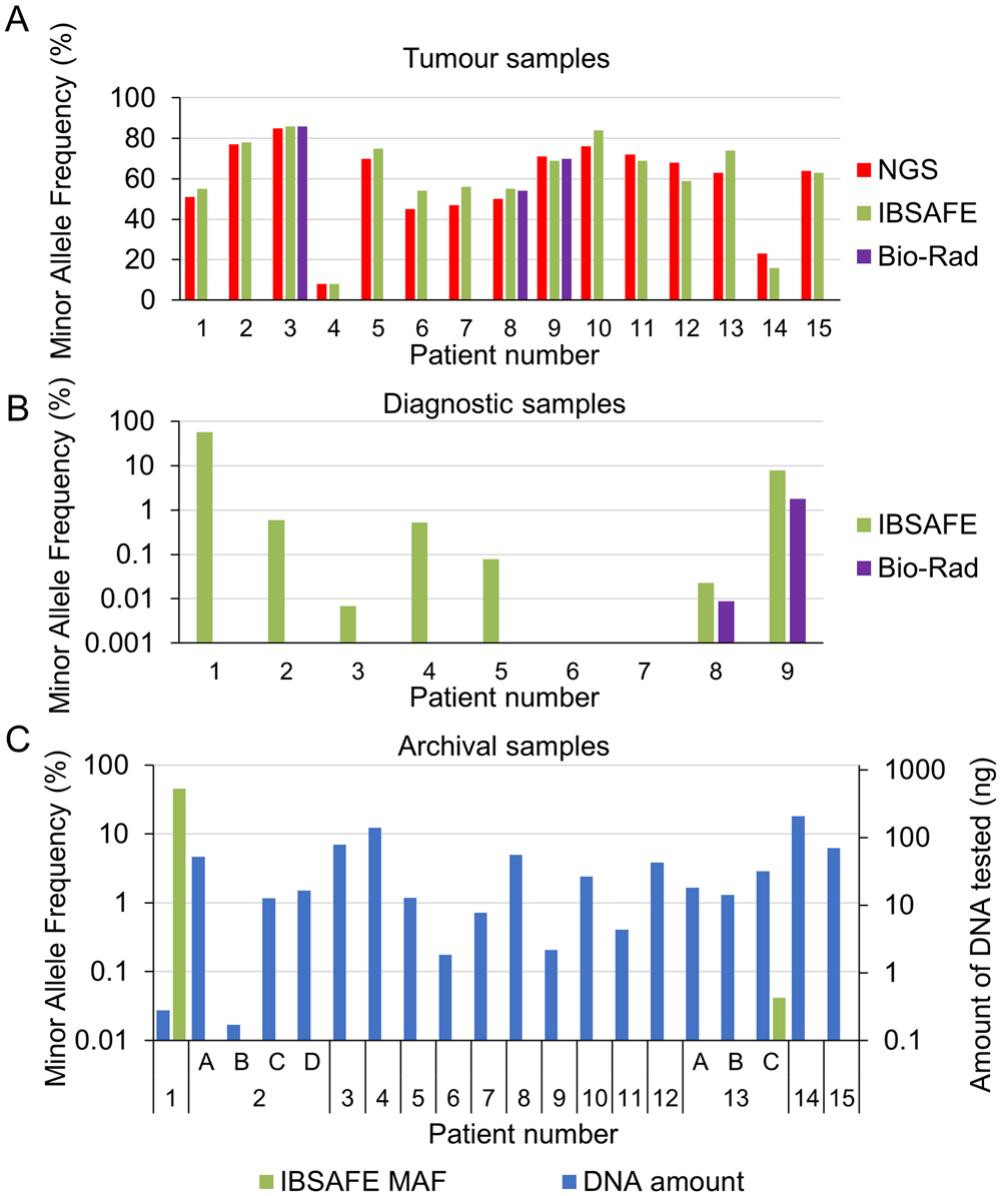
Minor allele frequencies (MAFs) (primary y-axis) for tumours (**A**), diagnostic samples (**B**) and archival samples (**C**). Amount of DNA tested (secondary y-axis) from archival samples (**C**). Note the log scale on the y-axis. Red, next-generation sequencing; green, IBSAFE assays; purple, Bio-Rad assays; blue: amount of DNA in ng.

We analysed both diagnostic and archival samples using IBSAFE, an ultra-sensitive ddPCR-based method. We were also able to analyse three diagnostic samples (from patients 3, 8 and 9) with the Bio-Rad-based ddPCR assay to serve as an additional control for the ddPCR approach. Using IBSAFE we were able to detect a tumour MAF comparable to that of the NGS approach (Table 3, Fig. 2A). The Bio-Rad assay performed equally well in the tumour setting. The measured MAFs of the IBSAFE method ranged between 8–86% with a median of 63%.

The IBSAFE method was able to detect tumour-identified mutations in seven of nine diagnostic samples, however one mutation was determined to be a germline event (see below); hence, true somatic mutations were detected in six of eight diagnostic samples. Furthermore, IBSAFE detected the tumour-identified mutation in diagnostic samples from two of three patients with stage II disease (patients 5 and 8). The Bio-Rad assay failed to detect any mutations in the diagnostic sample from patient 3 but was able to detect mutations in the diagnostic samples from patients 8 and 9 (Table 3, Fig. 2B). The calculated MAF in the diagnostic sample from patient 8 (0.0089%) was below the theoretical limit of detection of the Bio-Rad assay^21^.

Despite the age and the low abundance of DNA in the archival samples, the IBSAFE method was successful in all the individual assays, measuring concentrations comparable to the QUBIT assay (Tables 1 and 3, Fig. 2C). Furthermore, we were able to detect a tumour corresponding mutation in the archival samples from patients 1 and 13 (sample 13C). Patient 13 was diagnosed with a stage IIIB tumour and had a total of three archival samples (13A, B and C) collected 46, 32 and 20 months prior to diagnosis. A *TP53* mutation with a MAF of 0.042% was detected in sample 13 C obtained 20 months prior to diagnosis, with a total of 31.75 ng of DNA tested. The mutation was however not detectable in the earlier samples (13 A and B).

The MAF of 46% in the sample from patient 1 led us to suspect a germline *TP53* mutation, which was confirmed using a normal tissue sample (endometrial biopsy), from the same patient (data not shown).

No mutations were detected in the remaining archival samples (Table 3, Fig. 2C).

## Discussion

In this study we evaluated the ability of IBSAFE, an ultra-sensitive ddPCR method, to detect known *TP53* mutations in Pap samples from patients with HGSOC collected at the time of diagnosis and approximately two to seven years prior to diagnosis.

We were able to detect somatic *TP53* mutations in diagnostic samples from six of nine women using IBSAFE. *TP53* mutations were not detectable in two of the diagnostic samples, most likely due to the MAF being below the limit of detection of the IBSAFE method. The MAF estimates suggest that there is a huge variance in detectable somatic DNA mutations ranging from 0.0068% to 7.9%.

Importantly, IBSAFE was able to detect mutations with an in-sample limit of detection of 1 in 50,000. This sensitivity is much higher than that of conventional NGS^18–20,26^. Moreover, the MAF estimates of IBSAFE were higher than those of the commercial approach for the diagnostic samples, despite exhibiting similar MAFs in the tumour setting, possibly indicating higher in-sample sensitivity. This is probably due to the experimental set-up in which the Bio-Rad assay is optimised for 100 ng DNA inputs. In reality, DNA inputs from liquid biopsies, such as plasma and Pap samples, are limited and vary greatly between patients. Therefore, a method that relies less on the amount of DNA input will allow for an improved limit of detection in low abundance DNA samples. Of note, IBSAFE was able to perform in samples with as little as 0.17 ng of DNA input. However, due to the small number of samples tested no significance comparisons between the two ddPCR methods could be performed. Furthermore, a direct comparison of MAFs is complicated due to differences between the methodologies. Such a comparison would require a larger study containing also healthy controls and is beyond the scope of this proof-of-principle study.

Notably, we were able to detect a tumour corresponding mutation in an archival sample collected 20 months before diagnosis from a patient subsequently diagnosed with a stage IIIB tumour, while we did not detect any mutations in the two other samples collected at time points earlier than 20 months prior to diagnosis. Although the archival samples in our study were obtained within the suggested median time frame proposed by Labidi-Galy *et al*. (2017) and IBSAFE assays were successful in all the archival samples, we did not detect any *TP53* mutation in the remaining samples from the other women also obtained more than 20 months prior to diagnosis. We therefore speculate that the time-frame to detect HGSOC-derived mutations in Pap samples may be narrower. One explanation might be that although *TP53* precursor lesions have been suggested to be present approximately seven years prior to overt HGSOC, these precursors might not shed cells or DNA until later in the tumorigenic process. Importantly however, a 20-month window for early detection may confer a better prognosis for the patient, as suggested by the diagnosis in this case being stage IIIB HGSOC. Unfortunately, the limited number of patients in the present study precluded the possibility of exploring this further, and a larger study, with multiple archival samples per patient, collected at time-points closer to the time of diagnosis would be required to address this.

Although sequencing costs have been reduced during the last decade and population-wide screens for rare disorders have been suggested, deep sequencing is still not feasible for population-wide screens of genetically complex somatic diseases like cancer^27,28^.

Digital droplet PCR offers a clinically feasible platform, with ease of use, fast turnover and high sensitivity^21^. Although the assay sensitivity is high, one shortcoming of ddPCR is the limited number of mutations that can be detected in a single low abundance DNA sample. For ddPCR to be applicable in a clinical setting, attention should be given to collecting as much DNA as possible from liquid biopsies such as plasma or Pap samples. One such approach was illustrated by Wang *et al*., who evaluated a Tao brush for sampling in close proximity to the tumour^17^, which was found to improve the limit of detection. Other methods, such as a lavage of the uterine cavity have also been reported to improve detection of ovarian cancer^16^. However, for a diagnostic test to be successfully implemented in a screening setting, it is crucial that the sampling procedure imposes minimal stress to the patient.

Another limitation of conventional ddPCR is the ability to detect only a single mutation per assay. However, the emergence of multiplexing of ddPCR assays may allow for several mutations to be screened simultaneously^29^. This has recently proved efficient when genotyping *KRAS* mutations in non-small cell lung cancer^30^, and could possibly increase the utility of *TP53* screening even further, as mutations span the entire *TP53* gene^31^.

Although the size of the cohort limited the power of the present study, we were able to analyse both diagnostic and archival (pre-diagnostic) samples successfully despite low amounts of DNA. We were able to detect true somatic tumour corresponding mutations in diagnostic samples from six of eight patients. Of note, detection of low abundance *TP53* mutant DNA in Pap samples from two patients with stage IIA HGSOC emphasises the potential of the method for early detection. These findings may also support the use of such a sensitive method in the recurrent setting, where patients can be monitored for treatment response on a regular basis based on a known *TP53* mutation. Furthermore, we successfully detected a tumour-derived mutation in an archival sample collected 20 months prior to diagnosis from a non-symptomatic woman, and the IBSAFE ddPCR assay performed well in all archival samples. To our knowledge, this is the first time an ovarian cancer-derived mutation has successfully been identified in a pre-diagnostic Pap sample from a non-symptomatic woman.

Although the present study is based on a small number of patients, we believe that an ultra-sensitive ddPCR method should be evaluated in a larger cohort of patients with a greater number of serial pre-diagnostic samples collected prior to the time of diagnosis to provide a better resolution of the diagnostic potential of *TP53* testing in Pap samples for the pre-symptomatic detection of HGSOC.

## Methods

### Patients

We identified 79 women with HGSOC from an on-going study prospectively recruiting women with a suspected adnexal tumour at the Department of Obstetrics and Gynaecology in Lund at Skåne University Hospital in the southern Swedish healthcare region. Among these, liquid-based archival Pap samples collected before the ovarian cancer diagnosis were available from nine women from the cervical cancer screening program within the region of Malmö, Sweden at the Department of Medical Microbiology, Lund University Hospital, Sweden (cohort 1). Further, we identified an additional six women from whom matched liquid-based archival Pap samples and tumour tissue were available (cohort 2). All tumours were classified according to WHO 2014^32^ and staged according to the International Federation of Gynaecology and Obstetrics (FIGO) criteria^33^. The study was approved by the Ethics Committee at Lund University (Sweden) and was performed in accordance with the Declaration of Helsinki. The patients provided written informed consent.

### Sequencing of tumours

Tumour DNA was extracted using the AllPrep DNA/RNA Mini kit and blood DNA was extracted using the QIAmp DNA Blood Midi kit, both from Qiagen (Sollentuna, Sweden). Formalin-fixed paraffin embedded DNA was extracted using the QIAamp DNA FFPE Tissue Kit from Qiagen.

Paired tumour/blood samples were sequenced using the INVIEW Oncopanel All-in-one (Supplementary Table S1) and analysed using the in-house analysis pipeline at GATC (Ebersberg, Germany). Samples were aligned against the human reference genome hg19 (chromosomes only, UCSC) with Burrows–Wheeler Aligner (version 0.7.15). Local realignment was carried out using the Genome Analysis Tool Kit (GATK, version 3.7), and duplicate reads were removed using Picard (version 1.131). For one patient lacking paired normal DNA, a pooled reference genome was constructed from seven patients with available blood samples. One patient had two tumour samples, one from each ovary. Bam files were analysed using the Bcbio-nextgen pipeline (version 1.1.0) for paired tumour samples^34^, with Mutect2 from GATK^35^, Freebayes^36^, VarDict^37^ and Varscan2^38^ as mutation callers. Mutations were annotated using the Variant Effect Predictor^39^ and classified using the GEMINI framework^40^ to filter out possible germline mutations.

### DNA extraction from liquid-based Pap samples

Diagnostic samples were collected using a ThinPrep (Hologic Inc., Sollentuna, Sweden) brush at time of diagnosis and were kept in DNAgard (Sigma Stockholm, Sweden). Archival samples were collected using the BD SurePath liquid-based Pap test (Becton Dickson, Stockholm, Sweden) until 2014, after which ThinPrep was used. Following pathology review, residual materials were transferred to new tubes and centrifuged. Residual liquid was removed and the cell pellets were stored at −80 °C. Each cell pellet was resolved in 420 µl Specimen Transport Medium buffer (STM) (Qiagen, Hilden, Germany) and used for extraction of DNA with the QIAmp DNA Mini kit (Qiagen), using the manufacturer’s instructions but with two additional washes with each wash buffer. DNA was quantified using Qubit HS DNA kit (Thermo Fisher, Göteborg, Sweden).

### Droplet Digital PCR of Pap samples

All chemicals, primers and equipment were purchased from Bio-Rad (Solna, Sweden) and used according to the manufacturer’s instructions, unless otherwise stated. Primers for mutations were designed using Bio-Rad’s online tool (San Diego, CA, USA) (Supplementary Table S2).

Droplet digital PCR of diagnostic samples was performed using a QX100™ Droplet Digital PCR system (Bio-Rad) according to the manufacturer’s instructions. Briefly, a 22 µl PCR reaction was prepared for each sample using 100 ng of diagnostic sample DNA in a final concentration of 1x ddPCR Supermix with no dUTP, primers (450 nM), probes (250 nM), and restriction enzyme (HaeIII or MseI (5U), Thermo Fisher). Subsequently droplets were generated and transferred to a 96-well PCR plate (VWR, Spånga, Sweden). The plate was heat-sealed with pierceable foil (VWR), and amplification performed using a C1000 Touch deep-well thermal cycler (Bio-Rad). The cycling conditions were as follows: an initial denaturation cycle of 10 min at 95 °C, followed by 40 cycles of denaturation for 30 s at 94 °C, annealing for 60 s at 55 °C (ramping rate set to 2 °C/s), and a final incubation for 10 min at 98 °C, ending at 4 °C. The plate was transferred to the QX100 droplet reader and analysed using the automated settings of the QuantaSoft analysis software (Bio-Rad, Version 1.4.0.99). Patient-matched tumour DNA was used as a positive control and Human Genomic Female DNA (Promega, Nacka, Sweden) was used as a negative control.

### Detection of *TP53* mutations in archival samples using IBSAFE

Diagnostic and archival samples were analysed for their respective tumour *TP53* mutation using IBSAFE (SAGA Diagnostics, Lund, Sweden). IBSAFE utilises ddPCR droplets together with a proprietary methodology that allows for ultra-sensitive detection of mutations to a lower limit of detection of ~0.001% MAF. 120 ng of diagnostic sample DNA and varying amounts of archival sample DNA (0.17–206.14 ng) were analysed using IBSAFE by SAGA Diagnostics. IBSAFE reactions were performed in duplicate or quadruplicate. Patient specific tumour DNA (positive control) as well as normal Human Genomic DNA (Promega) (negative control) samples were included in every run to confirm assay performance: for all IBSAFE assays, zero false positive signals were present in the negative control analyses of at least 80,000 normal haploid human genome copies.

### Statistics

Statistical tests were performed in R (version 3.3.3) using a two-sided Mann-Whitney U test with a significance threshold of 0.05.

## Supplementary information

Supplementary tables 1 and 2

## Data Availability

All data, materials and results are kept at the Division of Oncology and Pathology, Department of Clinical Sciences, Skåne University Hospital, Lund University, Lund, Sweden and can be made available upon reasonable request to the corresponding author.
